# The British Journals: Midwifery

**Published:** 1839-04

**Authors:** 


### MIDWIFERY.

*Enormous weight of a Child at Birth.*

[This case is recorded by J. D. Owens, Esq., Surgeon, Haymoor, near Ludlow. The child was born dead.]

The weight and admeasurement of the child, as taken ten hours after delivery:—The long diameter, from the occiput to the root of the nose, 7¼ inches; the occipito-mental, 8⅔ inches; from the parietal protuberances, 5 inches; the circumference of the skull, 15¼ inches; the cirumference of the thorax over the xyphoid cartilage, 14⅓ inches; the breadth of the shoulders, 7¼ inches; the extreme length, 24 inches. The weight seventeen pounds twelve ounces.

*Lancet. Dec.* 22, 1838.

